# Strategies to improve the immunosuppressive properties of human mesenchymal stem cells

**DOI:** 10.1186/s13287-015-0178-y

**Published:** 2015-10-07

**Authors:** Myoung Woo Lee, Somi Ryu, Dae Seong Kim, Ki Woong Sung, Hong Hoe Koo, Keon Hee Yoo

**Affiliations:** Department of Pediatrics, Samsung Medical Center, Sungkyunkwan University School of Medicine, 50 Irwon-Dong, Gangnam-Gu, Seoul 135-710 Korea; Department of Health Sciences and Technology, SAIHST, Sungkyunkwan University, 50 Irwon-Dong, Gangnam-Gu, Seoul 135-710 Korea; Department of Medical Device Management and Research, SAIHST, Sungkyunkwan University, 50 Irwon-Dong, Gangnam-Gu, Seoul 135-710 Korea

## Abstract

Mesenchymal stem cells (MSCs) are of particular interest for the treatment of immune-related diseases because of their immunosuppressive capacities. However, few clinical trials of MSCs have yielded satisfactory results. A number of clinical trials using MSCs are currently in progress worldwide. Unfortunately, protocols and methods, including optimized culture conditions for the harvest of MSCs, have not been standardized. In this regard, complications in the ex vivo expansion of MSCs and MSC heterogeneity have been implicated in the failure of clinical trials. In this review, potential strategies to obtain MSCs with improved immunosuppressive properties and the potential roles of specific immunomodulatory genes, which are differentially upregulated in certain culture conditions, will be discussed.

## Introduction

Human mesenchymal stem cells (MSCs) can be isolated from a wide variety of tissues [1] and are promising candidates for cell-based transplantation and regenerative medicine therapies. Some of the unique features of MSCs that make them attractive targets for therapeutic applications are their tendency to preferentially home to damaged tissues, their unique immunosuppressive properties [2], their capacity for self-renewal, and their multilineage differentiation potential [3]. To date, more than 500 clinical trials involving the infusion or transplantation of MSCs have been registered at ClinicalTrials.gov, and about 20 % of them depend on the immunosuppressive properties of MSCs. Although the immunosuppressive properties of MSCs have been confirmed and most phase I clinical trials have not shown any biosafety issues, only modest outcomes have been obtained in further trial phases [4–6].

MSCs exhibit heterogeneity not only among donors but also according to the tissue from which they are isolated, such as adipose tissue and bone marrow (BM) [7–9]. Moreover, MSCs isolated from the same tissue of the same donor still tend to exhibit phenotypic and functional variability because of a lack of standardization in preparative protocols and culture methods [8, 10–12]. Therefore, it may be possible to enhance or suppress a certain function of MSCs by controlling their culture conditions. In this review, potential strategies to obtain MSCs with improved immunosuppressive properties and the potential roles of specific immunomodulatory genes, which are differentially upregulated in certain culture conditions, will be discussed.

## Mesenchymal stem cells

MSCs were first characterized by Friedenstein and colleagues, who identified an adherent, fibroblast-like cell population in adult BM [13, 14]. The International Society for Cellular Therapy (ISCT) provided three minimal criteria to define human MSCs with regard to their culture characteristics, biomarkers, and developmental potential [15]. First, MSCs must be plastic-adherent when maintained in standard culture conditions. Second, MSCs must express CD105 (SH2), CD73 (SH3/4), and CD90 and must not express CD45, CD34, CD14 CD11b, CD79α, CD19, or HLA-DR. Third, MSCs must differentiate into osteoblasts, adipocytes, and chondroblasts in vitro. These minimal criteria proposed by the ISCT to define human MSCs have been accepted and widely used by many investigators to characterize cells [15]. However, MSCs from different sources and donors and cultured under different conditions do not always behave in the same way in cell therapies, even though they meet the ISCT criteria [8, 16–20]. One possible reason for this discrepancy is that MSCs have many features (such as multipotency; variability of proliferation and migration potential; secretion of various cytokines, chemokines, and growth factors; and immunomodulatory functions) which are critical to exert their therapeutic effects; however, the ISCT criteria do not reflect these functional aspects of MSCs [8]. In fact, MSCs have the capacity to differentiate into multiple tissues, including bone, cartilage [21, 22], tendon [23], muscle [24], fat [25], and BM stromal connective tissue, the latter of which supports hematopoietic cell differentiation [26, 27]. In addition, MSCs have immunosuppressive properties and reduce inflammation, suppressing lymphocyte alloreactivity in vitro in mixed lymphocyte reaction assays [28, 29]. Intravenous administration of MSCs improves the outcome of neural [30] and lung [31] injury in experimental animal models primarily through paracrine effects and a shift from the production of pro-inflammatory to anti-inflammatory cytokines at the site of injury. MSCs exposed to interferon (IFN)-γ are activated and suppress graft-versus-host disease (GVHD) in vivo [2]. Thus, the immunosuppressive properties of MSCs may be able to repair tissue damage caused by the immune system in autoimmune-induced inflammatory bowel diseases such as Crohn’s disease [32] and ulcerative colitis [33], treat GVHD of the gut, liver, and skin after allogeneic hematopoietic stem cell (HSC) transplantation [34–36], and prevent the rejection of organ transplants [37, 38]. However, the detailed mechanisms underlying the therapeutic effects of MSCs, a heterogeneous population of ex vivo expanded cells [39–41], have not been fully elucidated.

## Heterogeneity of mesenchymal stem cells

MSCs vary tremendously in terms of phenotypic and functional characteristics such as their proliferation capacity, expression of several cell surface markers, and secretion of cytokines [7–10]. Interestingly, although MSCs have been continuously adapted in many laboratories, their heterogeneity is considered to be due mainly to the use of non-standardized culture protocols, including the starting material, culture media, levels of sera/cytokines/oxygen, number of passages, and cell density [7, 42, 43]. In this regard, Ho and colleagues [43] classified MSC heterogeneity as follows: (1) cellular heterogeneity of the initial population, (2) varied expansion capacity of specific subsets of cells and of the final population, and (3) long-term biological function of MSCs. In particular, ex vivo expansion of MSCs is used to develop and maintain MSCs for cell therapy, and the methods used to expand and characterize MSCs are critical for their preparation. Moreover, MSCs express a wide variety of cytokines, chemokines, and growth factors that are important for cell migration, homing, and immunomodulation, following reconstitution of damaged tissues [44–48]. Based on their functional effects, differences in the secretion of these molecules by MSCs may be critical for the outcomes of cell therapies. In this regard, it is important to identify the best subpopulation of cells, to determine how the cells are expanded and characterized ex vivo, and to determine when the cells should be used clinically.

Numerous attempts have been made to develop more specific procedures for the isolation and preparation of appropriate subsets of MSCs from a heterogeneous cell population [7, 11, 43]. The protocol most commonly used in preclinical and clinical studies to isolate MSCs from various tissues is centrifugation over a density gradient followed by ex vivo expansion, which removes hematopoietic cell contamination. With this method, cell recovery from each tissue is variable among operators, and technical expertise is required to consistently obtain MSCs with a high efficiency. In addition, numerous putative human MSC surface markers (i.e., CD49a [49], CD73 [3], CD105 [50], CD106 [51], CD271 [52], MSC antigen-1 [53], Stro-1 [54], and stage-specific embryonic antigen-4 [55]) have been identified thus far. These markers are used alone or in combination to enrich homogeneous MSCs and to avoid cellular contamination. Unfortunately, many of these markers are widely expressed in stromal cells and lack specificity, contributing to the significant heterogeneity among MSCs derived in a single isolation [56].

MSC culture variables include medium formulation, culture surface substrate, cell seeding density, physiochemical environment, and subculture protocols. In particular, the development of well-formulated culture media for the isolation and expansion of MSCs is imperative; however, this is as an extremely difficult process because of the high complexity of media formulations. In this regard, the disclosed medium formulations for MSCs (e.g., those reported in [57–60]) are best positioned to be further developed by the many investigators interested in the therapeutic applications of MSCs. Unlike some cell types, MSCs can survive in hypoxic environments for several days by upregulating survival pathways [61, 62] and increasing cellular metabolism [63]. Cell numbers are also increased when cells proliferate under low oxygen tension [64, 65]. Differentiation into different mesenchymal lineages can be enhanced by culture under some hypoxic conditions [66, 67]; however, the effects seem to depend on various variables such as the exact oxygen tension, time in culture, and use of hypoxic preculture. Moreover, hypoxic conditions enhance the paracrine role of MSCs by altering cytokine and growth factor release [68–70] and play an import role in mobilizing MSCs and recruiting them to sites of injury [69, 71, 72]. Thus, hypoxic preconditioning of MSCs prior to implantation and associated hypoxic conditioned medium can improve cell survival in vivo, which has significant effects on the long-term effectiveness of MSC therapy. However, protocols to prepare and characterize MSCs have not been standardized. If the heterogeneity of MSCs cannot be minimized, it might take a long time to produce satisfactory clinical results.

In recent years, preparing cell therapy products using MSCs often required complex procedures, such as multiple cell-selection steps, ex vivo expansion, cell activation (e.g., priming or licensing), encapsulation, and genetic modifications [73, 74]. These complex procedures reflect the increasing sophistication of cell therapies and their production methods but have also occurred in response to the potential risks and increasingly rigorous regulatory requirements for these novel cell therapies. In fact, among the methods described above, ex vivo expansion and cell activation may have only minimal regulatory issues in terms of their clinical application because ex vivo expansion is a general method used in cell culture and cell activation is a simple method in which cells are merely primed with cytokines such as IFN-γ, which are commercially available and approved by the US Food and Drug Administration for the treatment of several diseases [72, 75, 76]. However, there are many concerns regarding the use of engineered or modified cell therapy products for clinical applications, and appropriate solutions need to be developed in the near future.

## Modulating cell confluency to overcome mesenchymal stem cell heterogeneity

As previously mentioned, despite the well-known advantageous biological properties of MSCs, they have not been successfully adapted in clinical trials, because of the lack of standardized protocols, and this has resulted in their notorious heterogeneity. Many studies have examined the expansion capability and phenotypic properties of MSCs; however, the underlying mechanisms and the types of genes involved have been neglected. Among the various growth conditions, cell confluency is suggested to be a primary factor that can affect the characteristics of highly heterogeneous MSCs [77, 78]. MSCs have a better proliferation capacity when they are grown at a low confluency because this provides the space for cells to proliferate and means that nutrients and oxygen are shared by fewer cells [7, 42, 43]. A low initial plating density is considered to be beneficial for ex vivo MSC expansion [7–10]; however, most studies have investigated the effect of the initial plating density on the capability of MSCs to differentiate or expand in vitro, not on their biological functions in vivo [7, 11, 43]. By contrast, we reported that genes linked to immunity, defense, cell communication, signal transduction, and cell motility are more highly upregulated in MSCs harvested at a high confluency than in MSCs harvested at a low confluency [79, 80]. These reports also indicate that the immunosuppressive properties of MSCs are enhanced via complex pathways involving these upregulated genes. Thus, ex vivo expansion of MSCs and harvesting of MSCs at an adequate density could be a promising strategy to prepare MSCs for use in regenerative medicine.

### Cell proliferation-related genes are upregulated mainly in mesenchymal stem cells at a low density

Changes in the expression levels of cell proliferation-related genes during in vitro MSC culture are important for the capability of these cells to further proliferate and differentiate [78, 81, 82]. Previous studies suggest that a low initial plating density results in faster MSC expansion, leading to higher MSC yields [7, 11, 43]. Growth at higher densities is constrained by density-dependent growth inhibition; therefore, cells plated at a lower density have a higher doubling number per passage. Our gene expression profile data showed that 29 genes regulating proliferation, differentiation, and cell cycling activities were upregulated in MSCs harvested at a low confluency (50 %) but that only four of these genes were upregulated in MSCs harvested at a high confluency (>90 %) [79]. At a lower plating density, MSCs were dispersed evenly over the plate and rapidly filled the available space over time. As cells became confluent, their proliferation slowed because of cell-to-cell contact and this was reflected in the reduced expression of proliferation-related genes. Therefore, the improved proliferation rate of MSCs at a low confluency is due not only to the higher availability of space and conditions that increase the number of cells but also to the interactions of genes. However, the influence of gene expression levels on the proliferation potential of MSCs must be investigated further.

### Genes related to the functional characteristics of mesenchymal stem cells, including immunomodulation, are more highly expressed in high-density cultures

**Fig. 1:**
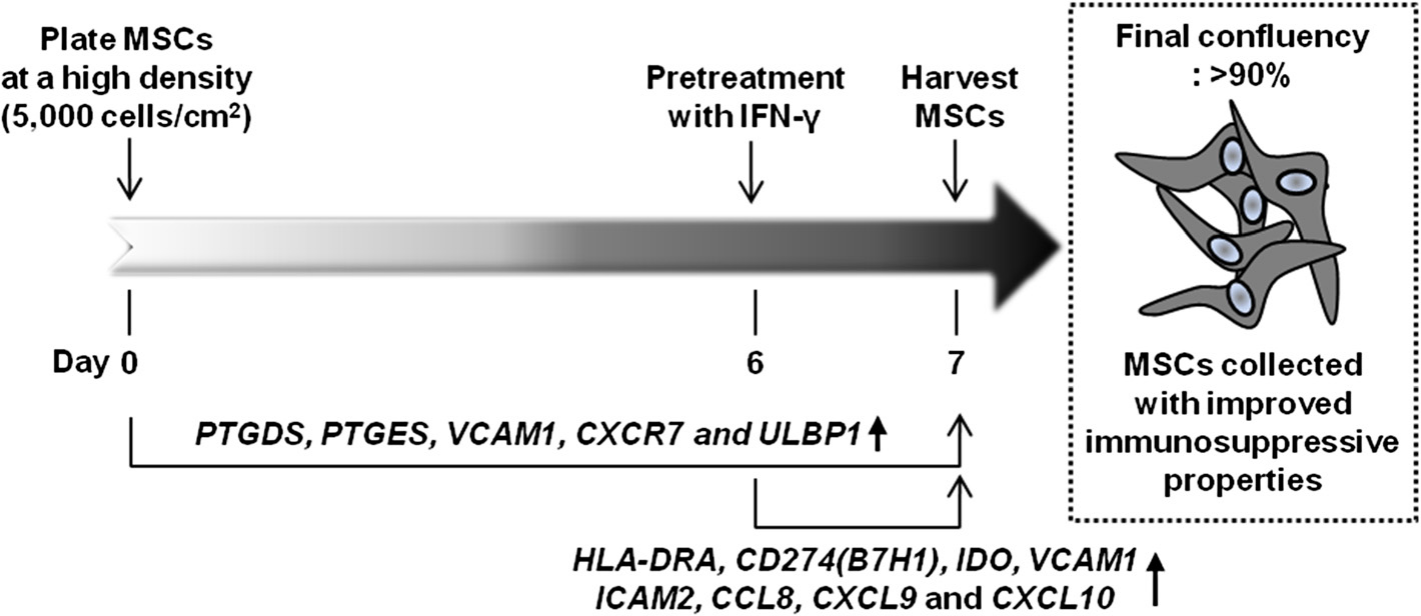
Strategies to obtain human MSCs with improved immunosuppressive properties. *CCL8* chemokine (C-C motif) ligand 8, *CD274(B7H1)* B7 homolog 1 (also known as programmed death-ligand 1), *CXCL9* chemokine (C-X-C motif) ligand 9, *CXCL10* chemokine (C-X-C motif) ligand 10, *CXCR7* chemokine (C-X-C motif) receptor type 7, *HLA-DRA* major histocompatibility complex, class II, DR alpha chain, *ICAM2* intercellular adhesion molecule 2, *IDO* indoleamine 2,3-dioxygenase, *IFN-γ* interferon-gamma, *MSC* mesenchymal stem cell, *PTGDS* prostaglandin D_2_ synthase, *PTGES* prostaglandin E synthase, *ULBP1* natural-killer group 2 member D ligand 1, *VCAM1* vascular cell adhesion protein 1

Our microarray analysis showed that a number of genes (276 of 24,526 genes) that control biological functions, including immunosuppression—such as prostaglandin D_2_ synthase (*PTGDS*), prostaglandin E synthase (*PTGES*), chemokine (C-X-C motif) receptor type 7 (*CXCR7*), vascular cell adhesion molecule 1 (*VCAM1*), and natural-killer group 2 member D (NKG2D) ligand 1 (*ULBP1*)—were highly upregulated in MSCs at a high confluency (Table 1 and Fig. 1) [79, 80]. These genes reportedly directly or indirectly (or both) affect the immunomodulatory activities of MSCs. Specifically, PTGDS and PTGES synthesize prostaglandin (PG) E_2_, which can stimulate or inhibit the activities of antigen-presenting cells (APCs), inhibit the proliferation of immature B cells and T cells, and regulate intracellular calcium release and p59(fyn) protein tyrosine kinase activity [83–85]. Although it remains controversial whether other types of PG function as pro- or anti-immunomodulatory molecules, PTGES is remarkable in terms of its immunosuppressive activities [84, 85]. The ability of MSCs to migrate, which is determined mainly by a panel of signals including chemokines, is closely related to the functions of MSCs in immune regulation and tissue repair [86]. CXCR7, which mainly activates mitogen-activated protein kinases and induces signaling following ligand binding, can regulate the immune system and inflammation via G protein-coupled receptors [87, 88]. Recently, many studies demonstrated that CXCR7, not chemokine (C-X-C motif) receptor 4 (CXCR4), mediates stromal cell-derived factor 1 (also known as CXCL12)-induced migration of various cells, including MSCs, and BM engraftment of cultured HSCs [89–92]. In addition, several studies have reported that MSCs can selectively migrate to specific injury sites after systemic infusion. This migration is mediated by very late antigen 4 (VLA4) and VCAM1, which allow MSCs to adhere to vascular endothelial cells and subsequently cross endothelial barriers [93, 94]. A certain type of APC assists the interaction of VCAM1 with VLA4 such that MSCs can migrate to inflammation sites and regulate T cell-mediated inflammation and pathology in non-lymphoid tissues [95–97]. On the other hand, ULBP1 is a ligand for NKG2D receptor, and the interaction of ULBP1 and NKG2D is reportedly essential for the delivery of activating signals to natural killer (NK) cells or the regulation of T-cell receptor-mediated activation of T cells or both [98–101]. We clearly showed the inhibitory roles of ULBP1 in the regulation of T-cell proliferation in vitro [80]. Furthermore, various cytokines (e.g., interleukin (IL)-15 and −17) are involved in signaling pathways in which ULBP1 takes part [102, 103], although definite mechanisms and signals regulating their activation still need to be elucidated. These highly expressed genes may be involved in the immunosuppressive properties of MSCs, and further studies of their precise roles in MSC functions are required.

**Table 1:** Gene Ontology analysis based on immunomodulatory function-associated genes upregulated in high-density MSC cultures (>90 %)

Gene Ontology Category_Biological Process	Total number of genes	Number of upregulated genes	Enrichment	*P* value
GO:0005576_extracellular region	21,529	91	17.89	<0.001
GO:0022610_biological adhesion	6813	37	8.31	<0.001
GO:0002253_activation of immune response	1360	6	3.87	0.016
GO:0016477_cell migration	5281	14	3.53	<0.001
GO:0042981_regulation of apoptosis	7116	26	2.1	<0.001
GO:0006955_immune response	6130	23	1.81	0.0012
GO:0050920_regulation of chemotaxis	598	3	1.79	0.084
GO:0048771_tissue remodeling	632	5	1.67	0.001
GO:0001558_regulation of cell growth	1805	8	1.32	0.033
GO:0042611_MHC protein complex	759	4	1.05	0.063

Microarray analysis was performed to compare the gene expression profiles of MSCs that were seeded at a density of 200 and 5000 cells/cm^2^, which were around 50 % and 90 % confluent on day 7, respectively. Upregulated genes in MSC cultures seeded at a density of 5000 cells/cm^2^ were sorted on the basis of gene expression profiles (fold change > 2 and *P* value < 0.05) and classified according to their related biological processes based on Gene Ontology terms by using DAVID Bioinformatics Resources 6.7

*MSC* mesenchymal stem cell, *DAVID* Database for Annotation, Visualization and Integrated Discovery

## Immunologically relevant effects of interferon-gamma

Increasing numbers of pre-clinical studies and clinical trials of MSCs to treat immune-related disease have shown encouraging results; however, the mechanisms underlying the immunosuppressive effects of MSCs need to be further investigated in order to effectively adapt these cells for therapeutic applications. The ability of MSCs to modulate the activity of surrounding cells is not constitutive but rather activated by signals from a pro-inflammatory environment [104]. This process, termed priming or licensing, is extremely complex, and little is known about all of the factors and signaling pathways involved [105]. Several reports have shown the roles of IL-1, IFN-γ, and tumor necrosis factor-alpha (TNF-α) in this process [76, 104, 106]. In particular, IFN-γ, the type II IFN, is a remarkable cytokine not only in innate and adaptive immunity to viral and bacterial infections but also in tumor control [107–111]. IFN-γ was initially believed to be secreted only by CD4 Th1 lymphocytes, CD8 cytotoxic T lymphocytes, and NK cells; however, other types of cells, including B cells, NK T cells, and IL-12-stimulated APCs, can also produce IFN-γ [107–109]. The main signaling pathway of IFN-γ involves JAK/STAT, although it also mediates the phosphatidylinositol-3 kinase/Akt pathway, phosphorylation of phospholipase C-γ2, and the extracellular signal-regulated kinase cascade, demonstrating its complex and widespread biological functions [109, 110]. The cellular effects of IFN-γ are notable because it can upregulate class I major histocompatibility complex (MHC), which is responsible for the host response to intracellular pathogens and the induction of cell-mediated immunity, and class II MHC, which promotes peptide-specific activation of CD4 T cells [108]. The immunosuppressive properties of MSCs are activated by other immune-related factors through IFN-γ, by itself or in combination with one of three pro-inflammatory cytokines, namely, TNF-α, IL-1α, and IL-1β. This has been demonstrated in a number of studies using GVHD in vivo models; the recipients of IFN-γ^−/−^ T cells do not respond to MSCs, and MSCs obtained from IFN-receptor-1-deficient mice do not possess immunosuppressive functions [2, 112]. In addition, after treatment with TNF-α and IFN-γ, MSCs are less effective at increasing pro-inflammatory cytokine production by activated peripheral blood derived-mononuclear cells and more efficient at inhibiting T-cell proliferation in an in vitro model [113]. TNF-α alone is sufficient to upregulate CXCR4 in MSCs in a time- and dose-dependent manner [114]. Lower expression of CXCR4 in MSCs leads to the failure of these cells to migrate into sites of inflammation and consequently reduces their immunosuppressive function [115]. Moreover, IL-1β released from monocytes enhances the secretion of transforming growth factor-beta by MSCs, which is involved in the inhibition of T-cell proliferation [104, 116]. In the presence of IFN-γ, either TNF-α or IL-1α induces the expression of intercellular adhesion molecule 1 (ICAM1) and VCAM1, which are also essential for MSC-mediated immunosuppression [117].

## Upregulation of immunomodulation-related genes following preconditioning of mesenchymal stem cells with interferon-gamma

We previously reported that activated T cells express higher levels of IFN-γ than quiescent T cells and that IFN-γ levels are significantly reduced when activated T cells are co-cultured with MSCs [118]. This is indicative of an IFN-γ autocrine–paracrine loop. Therefore, priming of MSCs with IFN-γ, in addition to harvesting highly confluent cells, was expected to produce promising outcomes with regard to enhancing the immunomodulatory properties of MSCs. Indeed, 512 of 24,566 genes were upregulated in IFN-γ-preconditioned MSCs (Table 2). Specifically, immunomodulation-related genes, such as MHC, class II, DR alpha chain (*HLA-DRA*) , *CD274* (B7 homolog 1 (*B7H1*)), indoleamine 2,3-dioxygenase (*IDO*), *VCAM1*, *ICAM2*, chemokine (C-C motif) ligand 8 (*CCL8*), chemokine (C-X-C motif) ligand 9 (*CXCL9*), and chemokine (C-X-C motif) ligand 10 (*CXCL10*), were dramatically upregulated by preconditioning MSCs with IFN-γ (Fig. 1). The three identified chemokines (C-C or C-X-C motif)—namely, CCL8, CXCL9, and CXCL10—play important roles in the recruitment of leukocytes leading to various immune responses, whereas the other genes tend to be more directly involved in the immunomodulatory properties of MSCs [119–121]. Specifically, *IDO*, a well-known IFN-γ-induced gene, was highly upregulated in IFN-γ-preconditioned MSCs, and IDO suppresses antigen-driven proliferation of T cells [122–124]. IFN-γ stimulated the expression of IDO in MSCs derived from sources other than BM, including human umbilical cord blood, adipose tissue, and Wharton’s jelly (a gelatinous substance derived from the umbilical cord) [118]. The immunosuppressive activities of IDO are mediated via the degradation of tryptophan, an amino acid that is essential for T-cell proliferation [123–125]. Thus, IDO and a paucity of tryptophan have received particular attention in many immune-related disorders. For example, patients with acute myeloid leukemia or adult T-cell leukemia/lymphoma were reported to exhibit a higher ratio of kynurenine (a tryptophan metabolite) to tryptophan than healthy subjects [126]. Therefore, the enhanced immunosuppressive activities of MSCs seem to be highly associated with IFN-γ priming and this is likely due in part to IDO induction. In addition, CD274 and HLA-DRA play a role during T-cell activation. HLA-DR encourages B7 subfamily members of immunoregulatory ligands to bind to T-cell receptors, and the B7H1 ligand (also known as CD274) inhibits T-cell responses [122, 127, 128]. In terms of in vivo adaptation, a few studies have revealed that BM transplantation of MSCs can control lethal GVHD, although this was not completely successful [129–131]. Furthermore, our unpublished study showed that mice injected with IFN-γ-pretreated MSCs had a higher rate of survival compared with those injected with untreated MSCs. Unfortunately MSCs are difficult to adapt as a first-line treatment for established GVHD because of the high cost and the lack of successful clinical data [132–134], and so MSCs harvested at a high density and preconditioned with IFN-γ may provide a solution to improve the results of future clinical trials (Fig 1).

**Table 2:** Gene Ontology analysis based on immunomodulatory function-associated genes upregulated in IFN-γ-treated MSC cultures

Gene Ontology Category_Biological Process	Total number of genes	Number of upregulated genes	Enrichment	*P* value
GO:0006952_defense response	9245	75	16.99	<0.001
GO:0006954_inflammatory response	2365	40	16.99	<0.001
GO:0009611_response to wounding	4854	49	16.99	<0.001
GO:0019882_antigen processing and presentation	1111	26	8.85	<0.001
GO:0006955_immune response	6130	39	8.85	<0.001
GO:0005125_cytokine activity	1242	22	5.85	<0.001
GO:0050863_regulation of T-cell activation	1122	17	3.74	<0.001
GO:0042981_regulation of apoptotic process	7118	44	3.58	<0.001
GO:0002819_regulation of adaptive immune response	536	10	2.73	<0.001
GO:0045088_regulation of innate immune response	852	9	2.33	<0.001

Microarray analysis was performed to evaluate the effect of IFN-γ pre-treatment on MSCs. Upregulated genes in MSC cultures pre-treated with IFN-γ were sorted on the basis of gene expression profiles (fold change > 2 and *P* value < 0.05) and classified according to their related biological processes based on Gene Ontology terms by using DAVID Bioinformatics Resources 6.7

*IFN-γ* interferon-gamma, *MSC* mesenchymal stem cell, *DAVID* Database for Annotation, Visualization and Integrated Discovery

## Strategies to obtain mesenchymal stem cells with enhanced immunosuppressive properties

In normal culture conditions, MSCs generally proliferate via cell division but rarely differentiate unless induced by particular differentiation conditions. Although low-density culture leads to faster proliferation, MSCs are initially plated at a lower density (200 cells/cm^2^) [79, 80, 82, 135] and it would take at least 2–3 weeks for MSCs to reach approximately 90 % confluency, as is required. Although high-density culture leads to slower proliferation, MSCs are initially plated at a higher density (5000 cells/cm^2^) and it takes an average of 7 days for cells to reach approximately 90 % confluency. In these cells, the expression of genes related to their function is increased [79, 80, 82, 136, 137]. These cells continue to proliferate, albeit slowly, and become over-confluent when proliferation is further induced. As a result, cells are damaged and transformed. For this reason, after 7 days of culture at a high density (5000 cells/cm^2^), cell confluency is around 90 % and MSCs can be obtained in which the expression of some genes related to immunosuppressive properties is increased. In addition, we believe that IFN-γ priming must be applied between 24 and 48 hours before MSCs are obtained for transplantation to use their improved immunosuppressive properties [76, 106, 113, 118]. Therefore, we suggest that low-density culture and thereby faster proliferation are needed to secure a large amount of MSCs and that MSCs must be cultured at a high density for 1 week before transplantation to maximize their immunosuppressive properties (Fig. 1). Furthermore, MSCs must be cultured according to the 1-week protocol, in which IFN-γ priming is applied between the fifth and sixth day and then cells are directly transplanted, to maximize the therapeutic effects of MSCs with improved immunosuppressive properties (Fig. 1).

## Conclusions

Cell confluency is of critical importance to produce functionally qualified MSCs for clinical uses; however, it is unclear how cell confluency at the time of harvest, not seeding, alters the expression levels of genes that regulate a specific biological function, such as immunomodulation. Although a low cell confluency effectively improves the in vitro expansion capacity of MSCs, the levels of immunomodulation-related genes are augmented in highly confluent MSCs. By contrast, many biological function-related genes showed a varied expression profile, representing the heterogeneity of MSCs. Thus, we presumed that an additional ex vivo treatment is required to overcome MSC heterogeneity; indeed, priming of MSCs with IFN-γ successfully improved their immunomodulatory functions. The expression profiles and functional analyses of specific genes presented herein suggest that MSCs with enhanced immunosuppressive properties can be produced by preconditioning MSCs that are almost confluent with IFN-γ. Therefore, these strategies are expected to provide useful guidelines for the collection of functionally qualified MSCs that can be readily adapted for further clinical uses, including therapies for immune-related disorders such as GVHD.

## Abbreviations

APC: Antigen-presenting cell
B7H1: B7 homolog 1
BM: Bone marrow
CCL8: Chemokine (C-C motif) ligand 8
CXCL10: Chemokine (C-X-C motif) ligand 10
CXCL9: Chemokine (C-X-C motif) ligand 9
CXCR4: Chemokine (C-X-C motif) receptor type 4
CXCR7: Chemokine (C-X-C motif) receptor type 7
GVHD: Graft-versus-host disease
HLA-DRA: Major histocompatibility complex, class II, DR alpha chain
HSC: Hematopoietic stem cell
ICAM1: Intercellular adhesion molecule 1
IDO: Indoleamine 2,3-dioxygenase
IFN: Interferon
IL: Interleukin
ISCT: International Society for Cellular Therapy
MHC: Major histocompatibility complex
MSC: Mesenchymal stem cell
NK: Natural killer
NKG2D: Natural-killer group 2 member D
PG: Prostaglandin
PTGDS: Prostaglandin D_2_ synthase
PTGES: Prostaglandin E synthase
TNF-α: Tumor necrosis factor-alpha
ULBP1: Natural-killer group 2 member D ligand 1
VCAM1: Vascular cell adhesion molecule 1
VLA4: Very late antigen 4
